# Multicenter retrospective evaluation of ileocecocolic perforations associated with diagnostic lower gastrointestinal endoscopy in dogs and cats

**DOI:** 10.1111/jvim.15731

**Published:** 2020-02-18

**Authors:** Vanessa L. Woolhead, Jacqueline C. Whittemore, Sarah A. Stewart

**Affiliations:** ^1^ Department of Clinical Science and Services Queen Mother Hospital for Animals, The Royal Veterinary College London UK; ^2^ Department of Small Animal Clinical Sciences University of Tennessee Knoxville Tennessee USA

**Keywords:** cecum, colon, colonoscopy, complication, ileoscopy, ileum, pneumoperitoneum

## Abstract

**Background:**

Ileoscopy is increasingly performed in dogs and cats with gastrointestinal signs, but iatrogenic ileocecocolic (ICC) perforations have not been described.

**Hypothesis/Objectives:**

To characterize endoscopic ICC perforations in dogs and cats.

**Animals:**

Thirteen dogs and 2 cats.

**Methods:**

This is a retrospective case series. Signalment, presentation, endoscopic equipment, colonic preparation, endoscopist's experience level, ileal intubation technique, method of diagnosis, perforation location, histopathology, management, and outcome data were collected and reviewed.

**Results:**

Six ileal, 5 cecal, and 4 colonic perforations were identified between 2012 and 2019. Dogs weighed 2.4‐26 kg (median, 10.3 kg) and cats 4.6‐5.1 kg (median, 4.9 kg). Endoscopy was performed in dogs presented for vomiting (n = 4), as well as large (n = 5), mixed (n = 4), and small (n = 1) bowel diarrhea. Cats had large bowel diarrhea. Endoscopists included 1 supervised intern, 9 supervised internal medicine residents (2 first year, 6 second year, 1 third year), and 5 internal medicine diplomates. Diagnosis was delayed in 5 dogs, occurring 1‐5 days after endoscopy (median, 3 days); dogs were presented again with inappetence (n = 4), lethargy (n = 4), abdominal pain (n = 3), retching (n = 2), and syncope (n = 1). All animals underwent surgical correction. Histopathology did not identify lesions at the perforation site in any animal. Two dogs required a second surgery; 1 died 12 hours after surgery. Survival to discharge was 93%, with 78% surviving ≥8 months.

**Conclusions and Clinical Importance:**

Iatrogenic endoscopic ICC perforation is not indicative of underlying disease and is associated with a good prognosis. Delayed diagnosis can occur. Therefore, perforation should be considered in the differential diagnosis for animals with clinical deterioration after endoscopy.

**Abbreviations**
ACVIM: American College of Veterinary Internal Medicine
AV: atrioventricular
CGS: Comparative Gastroenterology Society
ECVIM: European College of Veterinary Internal Medicine
ICC: ileocecocolic

## 1 INTRODUCTION

Endoscopy is used routinely to visualize the gastrointestinal mucosa and procure biopsy specimens from dogs and cats with a chronic history of gastrointestinal disease.^1^ Performance of ileoscopy is increasingly common because of discrepancies between duodenal and ileal histopathologic results.^2^, ^3^, ^4^, ^5^ Despite the increased frequency with which lower gastrointestinal endoscopy is performed, neither ileal nor cecal perforations have been reported, and none of the 4 reported colonic perforations occurred near the ileocecocolonic (ICC) valve.^6^, ^7^

Ileocecocolonic perforations occur in 0.01%‐0.16% of people undergoing diagnostic endoscopy.^8^, ^9^, ^10^, ^11^ Although most perforations are detected by direct visualization of a rent and abdominal viscera or persistent abdominal distension, 24% are diagnosed 24‐96 hours after endoscopy, typically because of persistent abdominal discomfort.^12^ Mortality after surgical correction ranges between 0 and 25%,^13^, ^14^, ^15^ with improved patient outcome for cases diagnosed immediately.^16^, ^17^

The purpose of our retrospective case series was to characterize ICC perforations occurring secondary to diagnostic lower gastrointestinal endoscopy in dogs and cats. Information was collected regarding the time and method of diagnosis, possible predisposing factors, treatment, and outcome.

## 2 MATERIALS AND METHODS

The American College of Veterinary Internal Medicine (ACVIM), the European College of Veterinary Internal Medicine (ECVIM), and the Comparative Gastroenterology Society (CGS) email list‐serves were solicited to recruit cases of iatrogenic ICC endoscopic perforation for this series. Medical records from contributing institutions were retrospectively reviewed by 1 of the authors (Vanessa Woolhead). Cases were excluded if therapeutic interventions were performed as part of the colonoscopy or if complete medical records were not available for review.

The following data were recorded for each animal enrolled: signalment, body weight, clinical signs and duration of the underlying intestinal disease, diagnostic and imaging test findings, colonoscopy preparation methods, adequacy of endoscopic visualization, ileal intubation technique, method and timing of perforation detection, clinical signs associated with endoscopic perforation, site of perforation, treatment, histopathological evaluation of biopsy specimens, and outcome. The experience level of endoscopist (intern, resident, resident‐trained, or diplomate), number of years of endoscopy experience, and endoscope and biopsy equipment used also were recorded. The adequacy of endoscopic visualization was retrospectively determined by the contributing endoscopist based on review of the endoscopy report and images obtained at the time of endoscopy. Adequacy was graded as good, satisfactory, poor, or nondiagnostic based on the extent of visualization of colonic mucosa: pristine, ≥50% of mucosal surface consistently visualized, <50% mucosa visible, and no visualization, respectively.

Categorical data are presented as percentages. Continuous nonparametric data are presented as median (range). The case series was approved by the Royal Veterinary College ethical review committee (URN SR2019‐0290).

## 3 RESULTS

### 3.1 Signalment and clinical presentation

Fifteen ICC perforations were identified by 7 university and 3 private referral hospitals between January 2012 and April 2019. Eight cases were reported through the ACVIM list‐serve, 1 each from the ECVIM and CGS list‐serves, and 5 from the authors' institutions. Perforations occurred in 13 dogs and 2 cats. Eleven dogs and both cats had undergone diagnostic endoscopy for clinical disease. The other 2 cases were healthy purpose‐bred dogs being used in a gastroenterology research study and endoscopy training course (1 each).

The median age of the dogs was 9 years and 11 months (range, 2‐14 years) and included 4 neutered females (31%), 3 intact males (23%), and 6 neutered males (46%). There were 3 cross‐breed dogs (23%), with 10 dogs of different breeds. Their median weight was 10.3 kg (range, 2.4‐26 kg). The cats were neutered males, aged 8 and 9 years, respectively. One was a Domestic Shorthair and the other a Turkish Van. The cats weighed 4.6 and 5.1 kg.

The most common clinical signs in client‐owned dogs prompting endoscopic examination were large (n = 5) or mixed (n = 4) bowel diarrhea, hematochezia (n = 3), and vomiting (n = 4). Weight loss was documented in 3 dogs, with concurrent hyporexia in 2 cases. Other clinical signs included small bowel diarrhea and abdominal pain (1 each). Clinical signs had been present for a median of 2.5 months (range, 3 weeks to 2.5 years; n = 9). Both cats were presented to the referral hospital for evaluation of large bowel diarrhea. One cat had weight loss with hyporexia, and the other cat had lethargy and weakness. Their clinical signs varied from 3 weeks to a few months in duration.

Serum biochemistry was evaluated in all animals except 1 research dog. Hypoalbuminemia (range, 1.8‐2.2 g/dL) was identified in 3 dogs and hyperglobulinemia in 1 cat. Abdominal ultrasound examination was performed in all clinical cases. Ultrasound examination identified mesenteric lymphadenomegaly in 2 dogs and 1 cat, and mucosal striations with irregular and thickened duodenal and colonic wall layering in 2 dogs. Abdominal ultrasound examination did not identify any abnormalities in the other 8 dogs and 1 cat. Serum cobalamin concentration was measured in 10 dogs and 1 cat; hypocobalaminemia was identified in 2 dogs.

### 3.2 Endoscopy

Perforation occurred during endoscopy performed by 1 supervised intern, 9 supervised internal medicine residents (2 first year, 6 second year, 1 third year), and 5 diplomates with 6‐20 years of endoscopy experience. Olympus GIF videogastroscopes were used most frequently (n = 12), with Fujinon and Storz endoscopes used in 1 case each. The endoscope used was unknown for 1 case. Endoscope insertion tube external diameter ranged from 5.5 to 11 mm (median, 8.6 mm; n = 13), whereas working length ranged from 103 to 133 cm (median, 103 cm). The biopsy forceps used were known in 7 cases, including 3 oval cups, 2 alligator jaw‐steps with a needle, and 1 each of serrated cup with or without a needle. Biopsy forceps were 2.3 mm in size in 4 cases, 1.8 mm in 1 case, and of unknown size for 2 cases.

Fasting time was unknown for 4 dogs and 1 cat. Dogs were fasted for a median of 48 hours (range, 12‐48 hours; n = 9). One cat was fasted for 12 hours. Eight dogs and 1 cat were given a mixed solution of polyethylene glycol 3350 and electrolytes PO the day before endoscopy; 1 dog was given bisacodyl. Ten dogs and 1 cat received at least 1 water enema, with 3 dogs also receiving a sodium dihydrogen phosphate dihydrate enema (Fletchter's Phosphate Enema; Purna Pharmaceuticals NV, Belgium). Upper gastrointestinal endoscopy was performed in 8 dogs and both cats. Colonic visualization was graded as good in 5 dogs and 1 cat (3 ileal perforations, 2 of which had delayed diagnosis; 2 colonic perforations, 1 of which had delayed diagnosis; 1 cecal perforation), satisfactory in 4 dogs and 1 cat (2 ileal and 2 colonic perforations; 1 cecal perforation that had delayed diagnosis), and nondiagnostic in 1 dog (cecal perforation). Visualization could not be retrospectively determined in 3 dogs (1 ileal perforation that had delayed diagnosis; 2 cecal perforations).

Direct endoscopic ileal intubation was performed in 4 dogs (3 ileal perforations, 2 of which had delayed diagnosis; 1 colonic perforation with delayed diagnosis), and 4 dogs had ileal intubation performed using biopsy forceps as a stylet (2 ileal and 2 colonic perforations). Blind ileal biopsies were attempted in 1 dog (cecal perforation with delayed diagnosis) and 1 cat (ileal perforation). Endoscopic ileal biopsy specimens were successfully obtained in 5 dogs and 1 cat. Endoscopic ileal biopsies were not attempted in 4 dogs and 1 cat because of diagnosis of a cecal (n = 4) or colonic (n = 1) perforation adjacent to the ICC valve.

### 3.3 Diagnosis and management

Iatrogenic ICC perforations were detected during or immediately after lower gastrointestinal endoscopy in 8 dogs and both cats. The perforation was directly visualized in 5 dogs and 1 cat. Persistent abdominal distension was noted at the end of the endoscopic examination in 2 dogs and 1 cat, and the endoscopist was unable to maintain gastrointestinal insufflation in 1 dog; all of these animals had pneumoperitoneum confirmed on abdominal radiographs obtained immediately after endoscopy while under general anesthesia.

**Figure 1:**
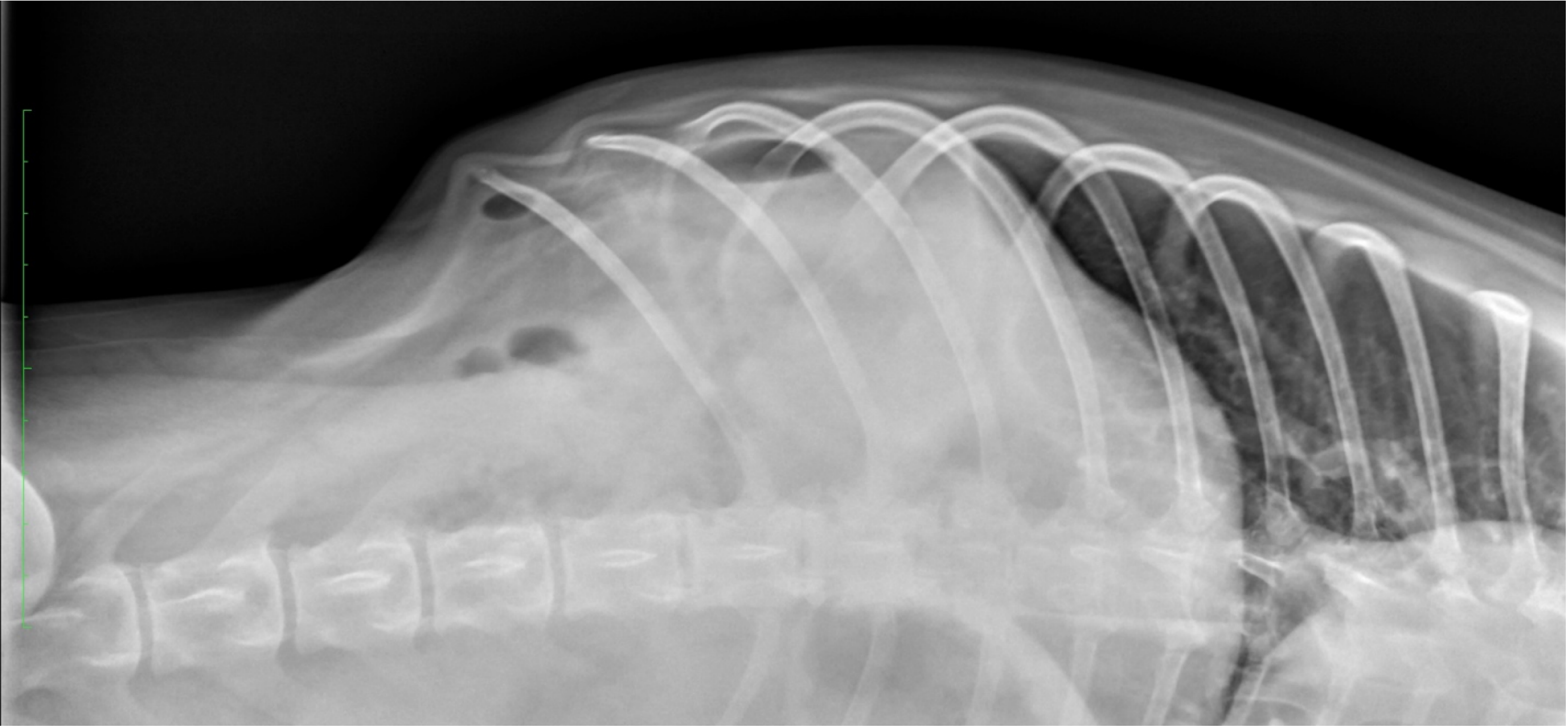
A horizontal beam ventrodorsal abdominal radiograph obtained from a dog 5 days after endoscopy demonstrating a pneumoperitoneum secondary to an iatrogenic endoscopic ICC perforation. ICC, ileocecocolic

Five of the 13 dogs experienced delayed diagnosis of ICC perforation. Delayed diagnosis occurred 1‐5 days after lower gastrointestinal endoscopy (median, 3 days); 4 dogs had been discharged after endoscopy and were presented again to the hospital because of deterioration in their clinical conditions. Clinical signs included inappetence (n = 4), lethargy (n = 4), retching and regurgitation (n = 2), and abdominal pain (n = 3); all dogs were normothermic. Two dogs were tachycardic, and 1 dog had cough and syncope associated with second degree atrioventricular (AV) block and high vagal tone. Abdominal ultrasound examination was performed in 3 dogs but identified the perforation initially in only 1 dog because of focal gas and fluid accumulation. Similar findings were found in a second dog but only on repeated ultrasound examination 2 days later, not at initial presentation. Radiographs were performed in 3 dogs (2, abdominal; 1, thoracic). Pneumoperitoneum was identified in all 3 dogs including the 1 dog without any ultrasonographic changes suggestive of perforation (Figure 1).

**Figure 2:**
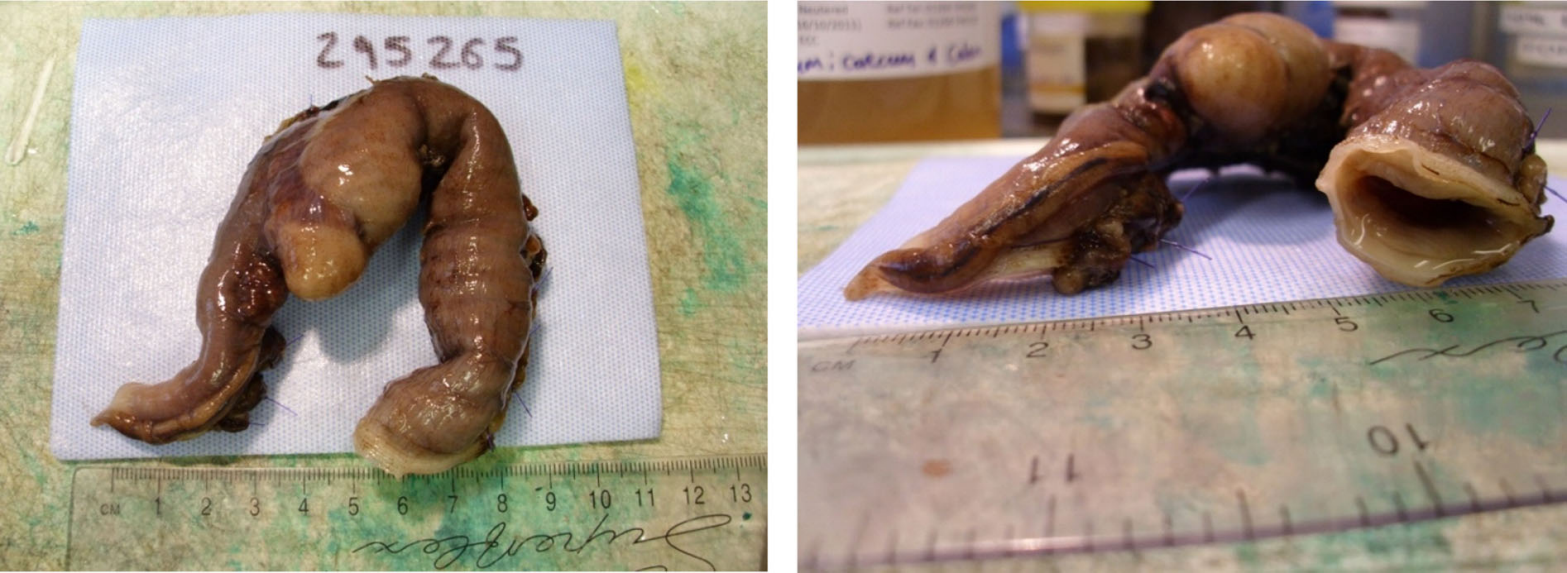
Photographs obtain of the ICC junction surgically removed by resection and anastamosis from a dog 5 days after endoscopy. The iatrogenic perforation is located on the mesenteric border of the colon adjacent to the ICC valve and is visible to the left and below the caecum. ICC, ileocecocolic

All animals immediately underwent exploratory midline celiotomy after diagnosis of ICC perforation. All perforations were adjacent to and within 3 cm of the ICC junction (Figure 2). Five dogs had an ileal perforation, with 3 on the mesenteric, 1 on the antimesenteric, and 1 between the mesenteric and antimesenteric borders. Four animals had colonic perforations located on the mesenteric border. Cecal perforation was identified in 4 dogs, affecting the mesenteric and antimesenteric borders in 1 dog each; the exact location of cecal perforation was not recorded for 2 dogs. Both cats had perforations on the antimesenteric border of the ileum and cecum. The approximate size of the perforation was recorded in 7 dogs and 1 cat. The median size of the perforation in animals diagnosed immediately was 7.5 mm (range, 1‐10 mm; n = 6), versus 1 and 5 mm in 2 dogs with delayed diagnoses. The putative source of the perforation was recorded in 11 cases as follows: endoscope tip (5 dogs), forceps‐induced trauma (3 dogs, 2 cats), repetitive ileal biopsy (1 dog). The perforation site was directly closed in 7 dogs and 1 cat, whereas 5 dogs had ICC resection and anastomoses performed; the closure technique was not documented in 1 dog and 1 cat.

Tissue from the perforation site was submitted for histopathology for 11 dogs and both cats. Histopathological findings were interpreted as changes secondary to perforation in all cases; no underlying pathological disease process was identified. Full thickness surgical biopsy specimens also were obtained from the same section of intestine but distant to the site of perforation in 5 dogs and both cats. Histopathology did not identify any abnormalities in 4 cases, whereas mild to moderate lymphoplasmacytic inflammation was present in the remaining 3. Additional regions of the gastrointestinal tract also were either endoscopically or surgically biopsied in 10 dogs and both cats, disclosing mild to moderate lymphoplasmacytic inflammation in 8 dogs and 1 cat, with concurrent lymphangiectasia in 2 dogs; histopathology did not identify any abnormalities in 2 dogs and 1 cat.

The 8 dogs and 2 cats that underwent surgical correction at the time of perforation all survived to discharge. Seven dogs were still alive at follow‐up after a median of 13 months (range, 1‐24 months). One dog was euthanized 48 months later for reasons unrelated to gastrointestinal disease. Both cats were alive at follow‐up 8 and 24 months later.

Of the 5 dogs that experienced delayed diagnosis of ICC perforation, 4 survived to discharge. Bacterial peritonitis was confirmed in 4 dogs, with 1 dog found to have a walled‐off abscess; bacterial culture was not performed in 1 dog. Two dogs required direct closure of the perforation, recovered uneventfully, and were alive at follow‐up 12 months later. Three dogs required resection and anastomoses. Of these, 1 dog died unexpectedly 2 weeks after surgery; the cause of death was not identified on necropsy. One dog required a second surgery because of persistent abdominal pain associated with omental adhesions and a sterile abscess; this dog was alive at follow‐up 11 months later. The final dog died after cardiopulmonary arrest 12 hours after having undergone a second surgery because of anastomosis breakdown.

## 4 DISCUSSION

Our retrospective case series documents 13 dogs and 2 cats that experienced endoscopic ICC perforation secondary to diagnostic ileocecocolonoscopy. Diagnosis was delayed up to 5 days in 33% of cases. The majority of animals had a good outcome after surgical correction, including 3 of 5 dogs in which diagnosis was delayed. Although seemingly rare, ICC perforation should be discussed with owners as a potential complication of ileoscopy when weighing the risks and benefits of the procedure. Endoscopic‐induced perforation should be considered as a differential diagnosis for animals with abdominal distention or clinical deterioration after endoscopy; abdominal radiography remains the imaging modality of choice for diagnosis.^18^, ^19^

Several risk factors have been associated with gastrointestinal perforation secondary to gastroduodenoscopy. Weight <10 kg is associated with increased risk of perforation in animals undergoing esophageal or gastric foreign body removal^20^ but this was not the case for ileocecocolonoscopy. Perforations also occur more commonly in cats, particularly those with ulceration and severe inflammatory or neoplastic gastrointestinal lesions^21^ but dogs were overrepresented in our study. Possible explanations for the higher number of perforations in dogs in our case series include less frequent performance of ileoscopy in cats because of equipment limitations, reticence in performing colonic preparation in cats, prioritization of full thickness biopsies in cats with muscularis thickening, and reporting bias.^22^ It is also possible that samples are collected less frequently from cats because of challenges in differentiating inflammatory bowel disease from small cell lymphoma, as well as similarities in their treatment and outcome.^21^, ^22^, ^23^, ^24^ Larger longitudinal studies will be required to determine the relative prevalence of ICC perforations in dogs versus cats.

Underlying disease was not identified at the site of perforation in any of the animals included in our series. In contrast, gastroduodenal endoscopic perforations are more frequently encountered in animals diagnosed with gastrointestinal lymphoma, marked inflammatory bowel disease or ulceration, suggesting that impaired gastrointestinal integrity might have been a contributing factor.^21^ Histopathologic abnormalities in our study were limited to mild to moderate inflammation at sites distant from the perforation, with lymphangiectasia found in only 2 cases. Neither neoplasia nor ulceration was identified, and the majority of animals had good long‐term outcomes.

In people, endoscopic ICC perforations are categorized as caused by either mechanical trauma or barotrauma; necrosis also is reported but only in association with therapeutic electrocautery.^12^, ^25^ Mechanical trauma refers to direct perforation of the wall with an object, such as the endoscope or forceps. Pneumatic‐ or barotrauma‐induced perforation refers to gastrointestinal rupture secondary to excessive insufflation without instrument‐wall contact. Although infrequent in people, it tends to occur where the gastrointestinal tract wall is thinner or impaired (ie, the cecum or an area of ulceration, respectively).^25^ Barotrauma previously was described in several cats with preexisting gastroduodenal ulceration^21^ but no animal was found to have mucosal defects in our case series. The endoscope tip or biopsy forceps was believed to be the source of the trauma in 13/15 animals in our study based on the sizes and locations of the perforations. However, a negative effect of excess insufflation on intestinal wall pliability, and thus resilience to mechanical contact, cannot be ruled out.

The experience level of the endoscopist or ability to adequately visualize the colonic mucosa also could contribute to the occurrence of perforations. Technical ability differs markedly between experienced and novice veterinary endoscopists,^26^ and gastroenterologists in human medicine are not considered competent at ileoscopy until after completion of extensive training, including intubating the ileum at least 50 times.^27^ In our series, the majority (n = 10) of perforations were experienced by interns or residents, which might have been influenced by reporting bias. The endoscopist's experience level also has been identified as a risk factor for perforation in some,^28^, ^29^ but not all, reports in human medicine.^12^, ^25^, ^30^ Our findings emphasize the importance of formal endoscopic training and careful supervision of novice endoscopists during ileoscopy.

Inadequate visualization has been identified as a possible cause for ICC perforation in studies in humans.^12^, ^16^, ^31^ Animals that experienced ICC perforation in our report were exposed to a variety of colonic preparation techniques. Although only 1 dog was deemed to have inadequate visualization of the colonic mucosa, grading of endoscopic visualization was performed by the endoscopist contributing each case. Furthermore, grading was not performed for 20% of cases. Future prospective studies that apply a standardized scoring system for adequacy of preparation will be necessary to determine the optimal pre‐endoscopic colonic preparation in animals, as well as assess its impact on the risk of iatrogenic perforation.

Ileal biopsies have been found to improve diagnosis of small bowel disease in dogs and cats, and thus ileoscopy is recommended as part of endoscopic characterization of diffuse small intestinal diseases.^3^, ^4^, ^5^, ^24^, ^32^ However, ileoscopy is a technically challenging procedure that might prolong the duration of general anesthesia and increase the risk of complications.^32^ Multiple techniques of obtaining ileal biopsy specimens were used in the cases described here, but no clear association was found among the endoscopic maneuver performed, the location, or the perceived cause of the perforation. Our findings support recent recommendations that the decision to pursue ileoscopy be made on an individual basis.^32^

Typically, endoscopists suspect endoscopic perforation if insufflation and visualization of the gastrointestinal mucosa cannot be maintained, the patient develops abdominal distension refractory to endoscopic suction, or the rent, peritoneum, or abdominal organs are visualized directly.^21^ Delay in diagnosis of gastrointestinal perforations is more common in people undergoing therapeutic versus diagnostic endoscopy. Such perforations usually are smaller, occurring secondary to delayed tissue necrosis and impaired colonic wall integrity after electrocautery.^25^ People undergoing diagnostic endoscopy more frequently develop larger perforations that are detected during the procedure,^14^, ^31^ but microperforations secondary to biopsy forceps can result in delayed diagnosis.^25^

Five dogs in our study experienced a delay in diagnosis of their perforations. Delayed diagnosis of rectal perforation secondary to therapeutic endoscopic interventions has been reported in 2 dogs.^33^ Animals experiencing delayed diagnosis in our case series developed vague signs including lethargy, inappetence, abdominal discomfort, and retching. One dog presented instead with syncope associated with second degree AV block, likely caused by high vagal tone. Interestingly, all animals were normothermic. Abdominal radiography was performed in only 2 of 5 dogs with a delayed diagnosis and was diagnostic in both cases. Perforation was diagnosed in a third dog by thoracic radiography that included the cranial abdomen. Ultrasonography detected free abdominal gas in only 1 of 3 dogs during initial evaluation, delaying diagnosis of ICC perforation. Our case series highlights the need to consider iatrogenic gastrointestinal perforation as a differential diagnosis and obtain abdominal radiographs for any animal that recovers in an unexpected manner or presents unwell in the days after endoscopy, despite having undergone the procedure with no reported complications.

All animals in our series underwent emergency surgical correction of intestinal defects. Primary perforation closure was more commonly performed (n = 8) and was successful in all animals, regardless of the defect size. Resection and anastomoses were necessary in 5 dogs, including 3 with delayed diagnosis; 2 dogs also required a second surgery because of intestinal wound dehiscence and persistent abdominal pain associated with omental adhesions. Surgical repair was associated with good outcome in all animals except 2 dogs; 1 died 12 hours after surgery and 1 died within 14 days of discharge. In several studies, morbidity rate increased in people when perforations were diagnosed >24 hours after endoscopy,^16^, ^31^, ^34^ but other studies found no association between the time to detection and patient prognosis.^14^, ^17^ The low number of cases in our series limits the ability to determine risk factors, but morbidity was higher in animals with delayed diagnoses. The overall outcome for animals experiencing iatrogenic endoscopic ICC perforation was very good, with 93% surviving to discharge and 78% surviving beyond 8 months, in comparison with animals with perforation secondary to gastroduodenal endoscopy where 86 and 57% survived to discharge and ≥8 months.^21^

Our study had several limitations. Case enrollment relied on endoscopists to both remember and volunteer information about complications they might have induced, a potentially sensitive topic. Thus, our report might underestimate the number of ICC perforations documented. Furthermore, because the majority of list‐serve recipients are specialists, endoscopic ICC perforations occurring in first‐opinion primary care veterinary practices were not identified for inclusion. Wide variability in institutional record‐keeping precluded determination of prevalence and potential risk factors. As with any retrospective case series, complete case follow‐up was not always available, and some data were incompletely documented within the medical records. Additionally, although considered extremely unlikely, alternative causes for perforation cannot be definitively excluded given that not all animals underwent abdominal imaging before or immediately after the procedure. However, findings uniformly supported acute inflammation and perforation, making naturally occurring occult perforation unlikely.

## 5 CONCLUSIONS

Iatrogenic ICC perforation is a seemingly rare but serious complication of diagnostic lower gastrointestinal endoscopy in animals. Perforation can occur in animals with normal gastrointestinal tracts and with minimal gastrointestinal disease. Consistent with prior reports, ICC endoscopic perforations might occur more commonly when performed by a novice endoscopist. Perforations are not consistently identified during endoscopy, and fever is uncommon. Orthogonal view abdominal radiographs should be obtained to rule out pneumoperitoneum in animals that deteriorate clinically after endoscopy. Diagnosis of ICC perforation can be delayed, but both survival to discharge and long‐term survival are good to excellent for cases that undergo surgical correction.

## CONFLICT OF INTEREST DECLARATION

Authors declare no conflict of interest.

## OFF‐LABEL ANTIMICROBIAL DECLARATION

Authors declare no off‐label use of antimicrobials.

## INSTITUTIONAL ANIMAL CARE AND USE COMMITTEE (IACUC) OR OTHER APPROVAL DECLARATION

The study protocol was approved by the Royal Veterinary College Ethics Committee (URN SR2019‐0290).

## HUMAN ETHICS APPROVAL DECLARATION

Authors declare human ethics approval was not needed for this study.

## References

[1] Slovak JE , Wang C , Sun Y , et al. Development and validation of an endoscopic activity score for canine inflammatory bowel disease. Vet J. 2015;203(3):290‐295. 25665921 10.1016/j.tvjl.2014.12.030

[2] Allenspach KA , Mochel JP , Du Y , et al. Correlating gastrointestinal histopathologic changes to clinical disease activity in dogs with idiopathic inflammatory bowel disease. Vet Pathol. 2019;56(3):435‐443. 30563436 10.1177/0300985818813090

[3] Procoli F , Motskula PF , Keyte SV , et al. Comparison of histopathologic findings in duodenal and ileal endoscopic biopsies in dogs with chronic small intestinal enteropathies. J Vet Intern Med. 2013;27(2):268‐274. 23398168 10.1111/jvim.12041

[4] Casamian‐Sorrosal D , Willard MD , Murray JK , Hall EJ , Taylor SS , Day MJ . Comparison of histopathologic findings in biopsies from the duodenum and ileum of dogs with enteropathy. J Vet Intern Med. 2010;24(1):80‐83. 20002555 10.1111/j.1939-1676.2009.0427.x

[5] Scott KD , Zoran DL , Mansell J , Norby B , Willard MD . Utility of endoscopic biopsies of the duodenum and ileum for diagnosis of inflammatory bowel disease and small cell lymphoma in cats. J Vet Intern Med. 2011;25(6):1253‐1257. 22092613 10.1111/j.1939-1676.2011.00831.x

[6] Jones BD . Endoscopy of the lower gastrointestinal tract. Veterinary Clinics of North America: Small Animal Practice. 1990;20(5):1229‐1242. 2238369 10.1016/s0195-5616(90)50302-2

[7] Leib MS , Baechtel MS , Monroe WE . Complications associated with 355 flexible colonoscopic procedures in dogs. J Vet Intern Med. 2004;18(5):642‐646. 15515578 10.1892/0891-6640(2004)18<642:cawfcp>2.0.co;2

[8] Shin DK , Shin SY , Park CY , et al. Optimal methods for the management of iatrogenic colonoscopic perforation. Clin Endosc. 2016;49(3):282‐288. 26888410 10.5946/ce.2015.046 PMC4895935

[9] Magdeburg R , Sold M , Post S , Kaehler G . Differences in the endoscopic closure of colonic perforation due to diagnostic or therapeutic colonoscopy. Scand J Gastroenterol. 2013;48(7):862‐867. 23697700 10.3109/00365521.2013.793737

[10] Chau T , Ng C , Lau L , Lai ST , Yuen H . Delayed mid‐ileal perforation secondary to acute‐on‐chronic ischaemia after a diagnostic colonoscopy. Scand J Gastroenterol. 1998;33(9):1002‐1004. 9759960 10.1080/003655298750027065

[11] Razzak I , Millan J , Schuster M . Pneumatic ileal perforation: an unusual complication of colonoscopy. Gastroenterology. 1976;70(2):268‐271. 1248689

[12] Panteris V , Haringsma J , Kuipers EJ . Colonoscopy perforation rate, mechanisms and outcome: from diagnostic to therapeutic colonoscopy. Endoscopy. 2009;41(11):941‐951. 19866393 10.1055/s-0029-1215179

[13] Park JY , Choi PW , Jung SM , Kim NH . The outcomes of management for colonoscopic perforation: A 12‐year experience at a single institute. Ann Coloproctol. 2016;32(5):175‐183. 27847788 10.3393/ac.2016.32.5.175 PMC5108664

[14] Teoh AYB , Poon CM , Lee JF , et al. Outcomes and predictors of mortality and stoma formation in surgical management of colonoscopic perforations: a multicenter review. Arch Surg. 2009;144(1):9‐13. 19153318 10.1001/archsurg.2008.503

[15] Hawkins AT , Sharp KW , Ford MM , Muldoon RL , Hopkins MB , Geiger TM . Management of colonoscopic perforations: a systematic review. Am J Surg. 2018;215(4):712‐718. 28865668 10.1016/j.amjsurg.2017.08.012

[16] La Torre M , Velluti G , Giuliani G , et al. Promptness of diagnosis is the main prognostic factor after colonoscopic perforation. Colorectal Dis. 2012;14(1):e23‐e26. 21831176 10.1111/j.1463-1318.2011.02755.x

[17] Park JH , Kim KJ . Management outcomes of colonoscopic perforations are affected by the general condition of the patients. Ann Coloproctol. 2018;34(1):16‐22. 29535983 10.3393/ac.2018.34.1.16 PMC5847398

[18] Shanaman MM , Schwarz T , Gal A , O'Brien RT . Comparison between survey radiography, B‐mode ultrasonography, contrast‐enhanced ultrasonography and contrast‐enhanced multi‐detector computed tomography findings in dogs with acute abdominal signs. Vet Radiol Ultrasound. 2013;54(6):591‐604. 23919809 10.1111/vru.12079

[19] Fitzgerald E , Barfield D , Lee KC , et al. Clinical findings and results of diagnostic imaging in 82 dogs with gastrointestinal ulceration. J Small Anim Pract. 2017;58(4):211‐218. 28276120 10.1111/jsap.12631

[20] Gianella P , Pfammatter NS , Burgener IA . Oesophageal and gastric endoscopic foreign body removal: complications and follow‐up of 102 dogs. J Small Anim Pract. 2009;50(12):649‐654. 19954441 10.1111/j.1748-5827.2009.00845.x

[21] Irom S , Sherding R , Johnson S , Stromberg P . Gastrointestinal perforation associated with endoscopy in cats and dogs. J Am Anim Hosp Assoc. 2014;50(5):322‐329. 25028434 10.5326/JAAHA-MS-5727

[22] Evans SE , Bonczynski JJ , Broussard JD , Han E , Baer KE . Comparison of endoscopic and full‐thickness biopsy specimens for diagnosis of inflammatory bowel disease and alimentary tract lymphoma in cats. J Am Vet Med Assoc. 2006;229(9):1447‐1450. 17078807 10.2460/javma.229.9.1447

[23] Marsilio S , Ackermann MR , Lidbury JA , Suchodolski JS , Steiner JM . Results of histopathology, immunohistochemistry, and molecular clonality testing of small intestinal biopsy specimens from clinically healthy client‐owned cats. J Vet Intern Med. 2019;33(2):551‐558. 30820999 10.1111/jvim.15455 PMC6430868

[24] Briscoe KA , Krockenberger M , Beatty JA , et al. Histopathological and immunohistochemical evaluation of 53 cases of feline lymphoplasmacytic enteritis and low‐grade alimentary lymphoma. J Comp Pathol. 2011;145(2–3):187‐198. 21333999 10.1016/j.jcpa.2010.12.011

[25] Martinez MTG , Poblador RA , Rapsos GL , et al. Perforation after colonoscopy—our 16‐year experience. Rev Esp Enferm Dig. 2007;99(10):588‐592. 18052662 10.4321/s1130-01082007001000005

[26] Usón‐Gargallo J , Usón‐Casaus JM , Perez‐Merino EM , et al. Validation of a realistic simulator for veterinary gastrointestinal endoscopy training. J Vet Med Educ. 2014;41(3):209‐217. 24947679 10.3138/jvme.0913-127R

[27] Iacopini G , Frontespezi S , Vitale MA , et al. Routine ileoscopy at colonoscopy: a prospective evaluation of learning curve and skill‐keeping line. Gastrointest Endosc. 2006;63(2):250‐256. 16427930 10.1016/j.gie.2005.09.029

[28] Lorenzo‐Zúñiga V , Moreno de Vega V , Domenech E , et al. Endoscopist experience as a risk factor for colonoscopic complications. Colorectal Dis. 2010;12(10 Online):e273‐e277. 19930145 10.1111/j.1463-1318.2009.02146.x

[29] Puchner R , Allinger S , Doblhofer F , Wallner M , Knoflach P . Complications of diagnostic and interventional colonoscopy. Wien Klin Wochenschr. 1996;108(5):142‐146. 8901128

[30] Anderson ML , Pasha TM , Leighton JA . Endoscopic perforation of the colon: lessons from a 10‐year study. Am J Gastroenterol. 2000;95(12):3418‐3422. 11151871 10.1111/j.1572-0241.2000.03356.x

[31] Iqbal CW , Cullinane DC , Schiller HJ , Sawyer MD , Zietlow SP , Farley DR . Surgical management and outcomes of 165 colonoscopic perforations from a single institution. Arch Surg. 2008;143(7):701‐706. 18645114 10.1001/archsurg.143.7.701

[32] Jergens AE , Willard MD , Allenspach K . Maximizing the diagnostic utility of endoscopic biopsy in dogs and cats with gastrointestinal disease. Vet J. 2016;214:50‐60. 27387727 10.1016/j.tvjl.2016.04.008

[33] Holt PE . Evaluation of transanal endoscopic treatment of benign canine rectal neoplasia. J Small Anim Pract. 2007;48(1):17‐25. 17212744 10.1111/j.1748-5827.2006.00254.x

[34] Kim HH , Kye BH , Kim HJ , Cho HM . Prompt management is most important for colonic perforation after colonoscopy. Ann Coloproctol. 2014;30(5):228‐231. 25360430 10.3393/ac.2014.30.5.228 PMC4213939

